# Recovery after Work: The Role of Work Beliefs in the Unwinding Process

**DOI:** 10.1371/journal.pone.0081381

**Published:** 2013-12-11

**Authors:** Zoe Zoupanou, Mark Cropley, Leif W. Rydstedt

**Affiliations:** 1 Department of Psychology, University of Surrey, Guildford, United Kingdom; 2 Lillehammer University College (HiL) ASV, Lillehammer, Norway; Catholic University of Sacred Heart of Rome, Italy

## Abstract

According to the Effort-Recovery model, mental or physical detachment from work is an important mechanism of work related recovery, as delayed recovery has been associated with range of negative health symptoms. In this paper, we examine whether recovery from work (in the form of mentally disengagement from work) is affected by the concept of ‘work ethic’, which refers to beliefs workers hold about their work and leisure and the effects of experiencing interruptions at work. Two indices of post-work recovery were utilized: problem solving pondering and psychological detachment. The study was conducted with 310 participants employed from diverse occupational sectors. Main effects of positive and negative appraisal of work interruption and beliefs were analysed using mediated and moderated regression analysis on problem-solving pondering and detachment. Weakened belief in wasted time as a partial mediator, reduced problem-solving pondering post work when interruptions were appraised as positive, and a high evaluation of leisure partially mediated problem-solving pondering when interruptions were appraised as positive. The results also showed that a high evaluation of centrality of work and leisure moderated the effect of negative appraisal of work interruption on elevated problem-solving pondering. Positive appraisal of work interruption was related to problem-solving pondering, and the strength of this association was further moderated by a strong belief in delay of gratification. In addition, employees' positive appraisal of work interruption was related to work detachment, and the strength of this association was further moderated by strong beliefs in hard work and self-reliance. These findings are discussed in terms of their theoretical and practical implications for employees who are strongly influenced by such work beliefs.

## Introduction

Post-work recovery is compromised when employees do not mentally disengage or ‘switch-off’ from work at the end of their working day. Some employees continue to think about work-related tasks or responsibilities during their leisure time. The Employment of Britain survey conducted among 3,000 workers revealed that 70% of them reported thinking about work issues/worries sometimes when not at work [1]. Evidence showed that 30% of workers ‘often’, very often' or ‘always’ think about work issues during their leisure time while 24% are irritated by their inability to ‘switch-off’ when not at work. The inability to switch-off from work, conceptualised as ‘work-related’ rumination, has been associated with a number of negative health issues including increased risk of cardiovascular disease [2], fatigue, and sleeping problems [3],[4],[5],[6],[7].

Using qualitative methodology, researchers have identified an over-arching ‘work philosophy’ theme, among high work ruminators who find it difficult to mentally unwind post-work [6]. Belief in the ‘centrality of work’, ‘hard work’ and ‘commitment to long hours of work’ were integral to this master theme. To date however, little attention has been paid to the role of core beliefs about work in the process of mentally unwinding from work.

Work beliefs have been linked to the ‘work ethic construct’ with particular emphasis on careful use of time and centrality of work [8]. ‘Work ethic’ is a multidimensional measure [8], [9] consisting of seven work values: centrality of work, delay of gratification, hard work, leisure, morality/ethic, self-reliance and wasted time. An important question for consideration is whether adherence to compelling work beliefs delays the unwinding process after work. In this study, we focus specifically on four core beliefs: (1) leisure, (2) centrality of work, (3) hard work and (4) delay of gratification. We examine their direct links to work rumination as well as their mediating and moderating roles in the relationship between work interruptions and work rumination.

## Theoretical Models Of Recovery From Work

The mechanisms facilitating the post work unwinding process involve work detachment and recovery. The Effort-Recovery model postulates that effort expended on work demands triggers load reactions such as psycho-physiological activation and behavioural reactions [10]. Researchers have shown that psychological detachment is a mechanism that assists the recovery experience [11]. They used the term psychological detachment from work to imply the ability of individuals to “switch-off” during off-job time by disengaging mentally or by ceasing to think and worry about work-related tasks [12],[11].

According to the Effort-Recovery model, psychological detachment implies that work tasks and activities no longer call upon the same human functional systems that are required at work. As discussed, recovery requires individuals to reduce or refrain from work demands during off-job time to allow their psycho-physiological system to return to its baseline [13]. The conservation of resources theory postulates that individuals who replenish their resources are able to recover and regain a positive mood [14], [15]. It refers to resources as “objects, personal characteristics, conditions, energies and financial assets that are important for an individual's survival” [15]. Drawing from both models, we argue that in order for recovery to occur, it is essential for individuals to detach both mentally and physically by not investing effort in using the same resources after work as those required during work.

### Interruptions at work

According to the Job-Demand-Control Model, job demands, including time demands, can result in strain if individuals fail to achieve work tasks on schedule [16]. Therefore, individuals are less likely to complete or achieve their goals if they are interrupted at work. Interruptions refer to “events that cause cessation and postponement of an ongoing activity” [17]. Because interruptions appear recurrently in everyday life, they interfere with task completion. Communication technology now makes work detachment more difficult, as individuals can remain connected to their job-related activities twenty-four hours a day via remote access to their computer, or via emails, and telephones [18].

Although some research has considered interruptions as welcome experiences, others highlight the negative effects of interruptions in work activity. For example, research found that interruptions prevented the completion of primary work tasks even if employees returned to it following interruptions [19]. In line with the Job Demand-Resources Model, continued job demands after work requiring the same physical and mental effort as during work leads to increased time demands and depletion of work resources, which in turn, results in exhaustion [20].

However, interruptions can also be classified as welcome distractions, particularly when a chat with colleagues provides a distraction from a boring and monotonous task [21]. An experimental study found that responses to interruptions were often considered welcome if the interruptions were perceived as being of a positive nature [17]. A study by the Basex showed that 94. 5% of knowledge workers regarded urgent interruptions caused by managers as acceptable, and 90. 8% of knowledge workers considered questions being raised by colleagues as acceptable [22]. This suggests that work interruptions are important, particularly when they provide the interrupted person with information necessary for the completion of work tasks.

### Work rumination and work beliefs

Research identified belief in the centrality of work as the main theme among high ruminators, who appeared emotionally and cognitively engaged in work and encountered difficulty in “switching-off” from work [6], [23], [24]. Other researchers postulated that unachievable goals are associated with rumination, depression and physical complaints and pose a major strain on individuals [25]. When work tasks are not completed throughout the day individuals who find it difficult to switch-off from work thoughts during leisure time have reported that they continue thinking about uncompleted tasks [6]. According to the ‘Zeigarnik effect’, individuals remember better the interrupted tasks because they have left them uncompleted [26]. Therefore, we reason that interrupted work tasks left uncompleted, increases the likelihood of individuals ruminating about work issues, post work.

Not all post work related thoughts are negative. For example, studies found that employees who focus their attention on solving work problems in their leisure time can improve their work performance [27]. Empirical evidence suggests that thinking about work issues during off-job time has some benefits, as it may result in a positive conception of work stressors [28], and provide distraction from a negative mood [28], [17]. Moreover, it was found that employees who generate positive thoughts about work during the weekend report less fatigue and exhaustion [29].

According to some researchers work rumination includes three types, which are conceptualized as affective rumination, problem-solving pondering and detachment [30]. However, the present paper is concerned with only problem-solving pondering and detachment. Problem-solving, more commonly referred to as problem-solving pondering, is defined as an individual's ability to reflect on positive events occurring at work or their search for solutions to work-related problems during off-job time [29],[30],[7]. It is characterized by the prolonged mental scrutiny of a particular problem or an evaluation of previous work in order to see how it can be improved, but it does not involve the emotional process that sustains arousal as in affective rumination [30], [7].

Employees of medical services who positively reflected on aspects of their work during the weekends reported a reduction of emotional fatigue after the weekend, increased ratings of social activities with friends, and increased rating of learning and health [29]. Similarly, clerical university employees who reported positive work reflection during vacation showed absence of health complaints, decreased levels of disengagement from work and increased ratings of task performance after their vacation. In contrast, employees who engaged in negative work reflection during vacation, reported increased ratings of exhaustion and increased effort on performing tasks when returned to work [31].

Others demonstrated that the narrative stories of individuals in different professions illustrated how their leisure time was used creatively to find solutions and new ideas to work-related problems while ‘escaping’ from work [32]. It was argued that work-related issues were retained in the subconscious mind, where information was processed during leisure periods. Specifically, this implied that creativity relating to work issues required an incubation period which included periods of hard work as well as leisure [32].

Affective rumination is another form of work rumination and is characterised by intrusive and pervasive thoughts about work, which are negative in affective terms [30], [7]. The more individuals attempt to suppress their pervasive thoughts out of consciousness, the more accessible they may become [33],[34], and this causes tension and annoyance. As a result, the intrusive thoughts about work affect the unwinding process as individuals remain emotionally and cognitively ‘switched on’ during their leisure time. Detachment from work is the counter element to intrusive and ruminative thoughts post work [30] and determines the ease with which individuals ‘switch-off’ and leave work behind. The concept of detachment refers to the individual's sense of being away from the work situation [35], [12]. In common parlance, psychological detachment is defined as ‘switching off’ from work demands or ‘forgetting’ about the working day [12]. Self-reported psychological detachment was found to be negatively related to job involvement [12], [36]. Psychological detachment, according to other researchers, was positively associated with positive mood and negatively related to fatigue [12].

The Protestant work ethic (PWE) was developed to measure work-related beliefs [9]. Researchers originally posited that the protestant ethic, despite its reference to religious belief, was the initial terminology for the term work ethic [37]. Max Weber conceived the work ethic as ‘a commitment to the value of hard work as the earning of money combined with strict avoidance of all spontaneous enjoyment of life’ [9] (pp. 71). In his book, *The Protestant Ethic and the Spirit of Capitalism*, Weber included the idea of puritan asceticism referring to ‘time should not be wasted’, ‘luxury should be denied’ and ‘pleasures should be delayed’. Weber suggested that capitalism should not incorporate religious beliefs, and this attitude caused a radical shift from protestant work ethic terminology to the term work ethic [38].

Researchers found that “protestant” work beliefs of leisure, delay of gratification and self-reliance were important predictors of task efficiency and job performance among employees, and that the centrality of work and careful use of time were important work ethic constructs [8]. However, one limitation of the available research to date is that there has been no attempt to establish a comprehensive range of factors to explain the process of unwinding from work. In light of this absence, the current study examines the extent to which work beliefs and the perception of the nature of interruptions at work are interrelated and whether they have an effect on the unwinding process.

**Figure 1:**
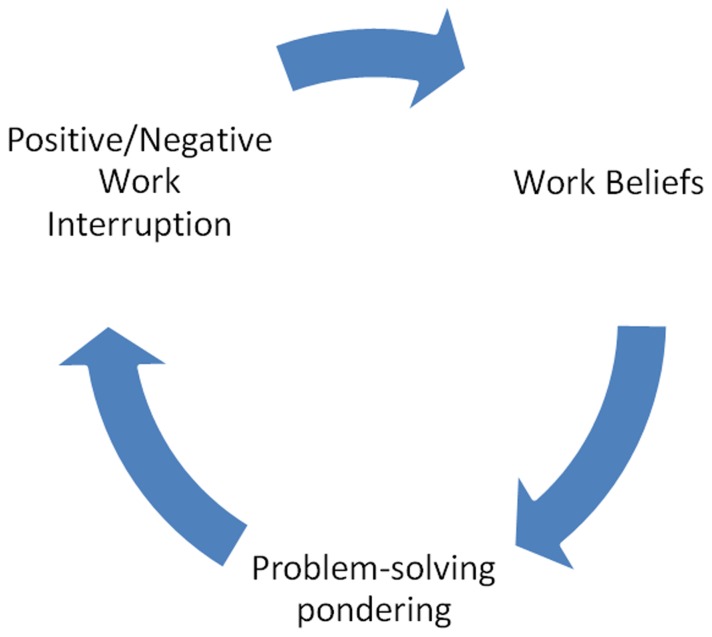
The role of work beliefs and work interruptions in problem-solving pondering.

*Hypothesis 1:* The“protestant” work ethic of morality/ethic, leisure, centrality of work and waste of time will be positively/negatively associated with problem-solving pondering (see Figure 1).

## Beliefs As Mediators In The Process Of Unwinding From Work Rumination

Previous research has focused on the extent to which work beliefs shape attitudes at work. It showed that individuals with the leisure ethic placed equal value on work time and recreation time [39]. Leisure ethic refers to recreation as work time was conceived to be meaningful only if leisure time was part of it. Individuals had the chance of pursuing activities during leisure time and work was conceived as a way of earning money. These authors also found that leisure beliefs, anti-work beliefs, wealth ethic and welfare beliefs were all interrelated.

In contrast, high work ruminators who experienced considerable high job strain after work showed a tendency towards work-related ruminative thinking [40]. Ruminative thinking during leisure time has been associated with delayed sleep onset [3], [4], [5]. Other studies showed that work-related ruminative thinking in the evenings triggered high autonomic arousal and delayed sleep onset [5], [41]. Ruminators who endorsed beliefs in hard work and long working hours were particularly likely to report impaired sleep [40]. ‘Centrality of work’ was also prominent among high ruminators who had difficulty in cognitively detaching from work, causing work to monopolize their life [6], [23], [24].

As evidence in support of a mediating role of work beliefs in the process of problem-solving pondering remains scarce, more refined research in this area is needed. Individuals who are fully engaged in work, experience high levels of positive affect [42] and thus have more cognitive and emotional resources [15] to cope with challenging events including work interruptions. We argue that individuals who appraise interruptions in a positive way are willing to invest effort in the face of interruptions to resolve problems and are also able to disengage from work issues during off-job time. A study showed a positive relationship between day-level recovery before work and day-level work engagement before leaving the workplace [42]. Relaxation during leisure allows individuals to reflect positively on the good sides of their work [43]. This positive work reflection was associated with the generation of creative ideas at work and personal initiative such as tackling, attacking and solving problems at work. Recovery experiences of psychological detachment and relaxation during the weekend were positively associated with weekly task performance [44]. This suggests that highly recovered individuals have resources that can be allocated to work tasks during the week. By contrast, we argue that individuals who face work interruptions and at the same time hold strong beliefs in the importance of efficient use of time, refrain from problem-solving pondering outside of work, as they prioritize work tasks during work, whereas strong positive evaluation of leisure could prolong the time spent on problem-solving pondering. Therefore, we argue the following:

*Hypothesis 2:* Belief in the importance of efficient use of time mediates the relationship between appraisal of work interruptions and reduced problem-solving pondering.

*Hypothesis 3:* Belief in leisure mediates the relationship between appraisal of work interruptions and increased problem-solving pondering.

## Beliefs As Potential Moderators

There is empirical evidence that some work beliefs moderate between work and psychological distress [45]. A study showed that strong belief in hard work was related to lower psychological distress and increased well-being [45]. Anti-leisure beliefs were associated with good time management and time structure. However, we reason that interruptions at work will be associated with greater problem-solving pondering during off-job time.

Employees who leave work tasks uncompleted due to interruptions and who have strongly held work beliefs about centrality of work may experience high emotional and cognitive activation levels. According to the job-demands-resources model, job demands inhibit recovery as they lead to exhaustion [20], [46]. Thus, to promote well being, employees should reduce job demands that require sustained cognitive and emotional effort. This leads to our fourth and fifth hypotheses:

*Hypothesis 4:* Belief in leisure moderates the relation between negative interruption and increased problem-solving pondering. In more detail, this relation will be stronger for a weak belief in leisure than for a strong belief in leisure. Belief in centrality of work moderates the relation between negative interruption and decreased problem-solving pondering. This moderating effect will be stronger for a weak belief in centrality of work than for a strong belief in centrality of work.

*Hypothesis 5:* Belief in delay of gratification moderates the relation between positive interruption and increased problem-solving pondering. This moderating effect will be stronger for a weak belief in delay of gratification than for a strong belief in delay of gratification.

On the basis of the Effort-Recovery model, employees who psychologically detach from work are able to restore lost energy and renew resources [10]. In addition, when employees perceive work interruptions as positive and have strong beliefs in hard work, psychological detachment from work during off-job time is probable; employees are likely to stop thinking about work-related issues when tasks have been completed at work. This leads to the sixth hypothesis:

*Hypothesis 6:* Beliefs in hard work and self-reliance moderate the relation between positive interruptions and decreased detachment from work rumination. The moderating effect on reduced detachment from work is stronger for employees with weak belief in hard work than for employees having strong belief in hard work. Similarly, the moderating effect on reduced detachment from work is stronger for employees with weak belief in self-reliance than for employees having strong belief in self-reliance.

## Methods

### Ethics Statement

The study was carried out in accordance with ethical guidelines of the University of Surrey and the British Psychological Society. Based on Faculty of Arts and Human Sciences Ethics criteria of the University of Surrey, this study did not require formal ethics procedure (see http://www. surrey. ac. uk/fahs/files/Ethics). The data was generated from primary resources (questionnaires) that did not include offensive wording and the research participants were not considered vulnerable. Issues of confidentiality and anonymity (of the data) were guaranteed. Participants were requested to give written consent to participate and could withdraw from the study if they wished.

### Participants

The sample included (*N = 310*) white-collar employees mainly from the private business sector. They were recruited from a range of organizations: 73% worked in customer marketing services, 20% held managerial positions and 7% worked in the accounting and executive sector; 50% were male (*N = *155) with a mean age of 35 years (*SD = *10. 7). The females (*N* = 155) had a mean age of 32 years *(SD* = 10. 5*)*. All employees worked full time with a mean of 40. 6*(SD* = 14. 4*)* working hours per week.

### Measures

#### Work interruptions

To assess interruptions at work, we generated items from a review of literature and conducted focus group interviews with white-collar workers. Work interruptions were measured with 13 items. In respect of content validity, two conceptually inconsistent items were deleted from the work interruption measure. Thereafter, the new scale was administered to another sample of employees. A 5-point Likert scale was used for the responses (1 =  strongly disagree; 5 =  strongly agree) (e.g., ‘I find being interrupted at work is a welcome distraction’, ‘It does not trouble me to leave work tasks unfinished at the end of the day’, ‘Interruptions can be a welcome break’, and ‘Interruptions reduce boredom’). The 11 items of the Interruption scale were subjected to principal component analysis, which revealed the presence of two components with Eigen values exceeding 1, accounting for 55.16% of the total variance. Seven items were loaded on factor 1 (labelled negative interruption) and three items loaded on factor 2 (labelled positive interruption). The Cronbach's alpha for the negative interruption was 0. 85 (*M = 16. 44, SD = 3. 12*), and for the positive interruption 0. 78 (*M = 10. 79, SD = 1. 57*). The correlation between the two factors was not particularly high (*r* = . 35).

To assess post work ruminative thinking, two sub scales (Problem-solving pondering and detachment) from the *Work-Related Rumination Questionnaire (WRRQ)*were used [47], [7]. These items were rated on a 5-point Likert-type scale (1 =  Very seldom or never; 5 =  Very often or always). The Cronbach's alpha for problem-solving pondering was .80, and .83, for detachment.

#### Stressor Question

Work stress was assessed by the single item “How do you find your job?”. This item was previously used in the Bristol Stress Study [48]. The item was rated on a 5-point scale (1 =  *Not at all stressful*; 5* = extremely stressful*).

#### Multidimensional work ethic profile measure (MWEP) [8]

The MWEP comprises of 64-items that are rated on a 5-point Likert-type scale (1 =  Strongly agree; 5 = Strongly disagree), with a lower degree score indicated greater belief in work. The MWEP supported seven dimensions: Centrality of work, Delay of gratification, Hard work, Leisure, Morality/Ethics, Self-reliance and Wasted time. The internal consistency (Cronbach's alpha) of the factors was good: wasted time (*α* = .72, 7 items), delay of gratification (*α* = .82, 7 items), centrality of work (*α* = .82, 10 items), hard work (*α* = .86, 10 items), leisure (*α* = .89, 10 items), morality/ethics (α = .78, 10 items). The morality/ethics item was recoded using a Likert-type scale (5 =  Strongly agree; 1 =  Strongly disagree).

### Data analysis

Mediated and moderated regression analysis, according to Baron and Kenny [49], was used to test the direct, indirect and interaction effects of problem-solving pondering and detachment on negative/positive work interruption. The Sobel test was used to test for mediation. The multiple regression (R) and the correlation analysis tests are conducted at the 0. 01 level. The sample size for power is .80.

## Results

Means, standard deviations, and correlations of all the study variables are presented in Table S1. As shown, the inter-correlations indicate first that positive and negative work interruptions were associated with the dimensions of work rumination. Problem-solving pondering was positively correlated with negative interruption *(r = .16)* and detachment was negatively correlated with negative interruption(*r = *−.33). Furthermore, problem-solving pondering was positively correlated with positive interruption (*r* = .19) and detachment was negatively correlated with positive interruption *(r = .*−*26)*. Moreover, the two forms of work interruptions were significantly positively correlated with each other(*r* = .38).

Problem-solving pondering has positive correlations with morality/ethic (*r* = .22) and leisure (*r* = .21) and negative correlations with centrality of work (*r* = −.21) and wasted time (*r* = −.18) *(Hypothesis 1)*. The two dimensions of work rumination; detachment and problem-solving pondering were significantly correlated (*r = *−.*51*).

### Mediating effects of Wasted Time

For problem-solving pondering, the mediating effect emerged indirectly(0. 02) through a weak belief in wasted time, *Sobel Z* = 1. 93, *p*<0. 05 (Table S2) *(Hypothesis2)*. With wasted time in the equation, the unstandardised regression coefficient for negative interruption on problem-solving pondering is reduced from 0. 27 to 0. 23. Consequently, there is support for the assumption that a belief in the importance of efficient use of time partially mediates the relationship between negative appraisal of work interruption and problem-solving pondering. The results of the regression analysis in Table S2 show the proportion of negative interruption (0.05) on problem-solving pondering, consisting of the direct effect (0.13) and the indirect effect through wasted time (0.02). Therefore, the belief in the importance of efficient use of time as a mediator accounts for 2% of the total effect of negative interruption on problem-solving pondering. Moreover, for multiple partial correlations, an *f* of .07 is considered a medium effect size [50]. For the regression, an *R^2^* of 0.05 yields a *f^2^* of 0.07, which is considered a medium effect size.

### Mediating effects of Wasted Time and Leisure

For problem-solving pondering, the mediation effect is indirect (0.03) through the belief in the importance of wasted time, *Sobel Z* = 2. 02, *p*<0.05 *(Hypothesis 2)*. The results of the regression analysis in Table S2 show the proportion of positive interruption (0.06) on problem-solving pondering, consisting of the direct effect (0.16) and the indirect effect through belief in wasted time (0.03).

Further, for problem-solving pondering, the mediation effect is indirect *(0.02)* through leisure attitudes, *Sobel Z* = 2. 33, *p*<0. 01 (Table S2) *(Hypothesis 3)*. The results of the regression analysis in Table S2 show the proportion of positive interruption (0.07) on problem-solving pondering, consisting of the direct effect (0. 16) and the indirect effect through leisure attitudes (0.02). Thus, there is support for the assumption that attitudes towards leisure and wasted time partially mediate the relationship between positive appraisal of work interruptions and problem-solving pondering. In summary, Hypotheses 2 and 3 are supported by the data.

In sum, although all the indirect effects are significant, the most substantial mediation effect was with leisure as an accountable mediator.

### Moderating effects of Leisure and Centrality of Work

Tables S3, S4 and S5 display the significant interaction effects between attitudes towards negative interruption and leisure *(ß = −0. 11, p<0. 05)*, negative interruption and centrality of work *(ß = 0. 13, p<0. 05)*, positive interruption and delay of gratification *(ß = −0. 15, p<0. 01)* on problem-solving pondering. Following Aiken and West, mean centred data was used [51].

**Figure 2:**
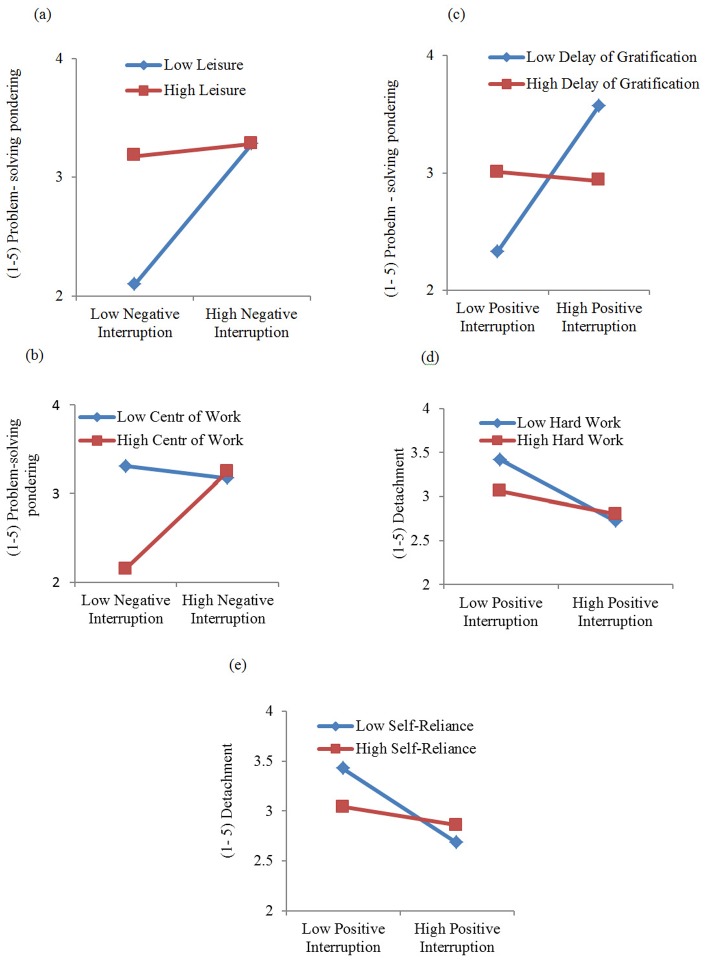
The relationships between work interruption and problem-solving pondering; and work interruption and detachment. (a). The relationship between work interruption and problem-solving pondering as a function of belief in leisure. (b): The relationship between work interruption and problem-solving pondering as a function of belief in centrality of work. (c): The relationship between work interruption and problem-solving pondering as a function of belief in delay of gratification. (d): The relationship between work interruption and detachment as a function of belief in hard work. (e): The relationship between work interruption and detachment as a function of belief in Self-reliance.

Graphical representations of the interactions are shown in Figure 2.

Figure2a shows elevated problem-solving pondering under conditions of weak belief in the importance of leisure generally but especially under high negative interruption. Moreover, the combination of high negative interruption with weak belief in centrality of work (see Figure 2b) was shown to be associated with lower problem-solving pondering. Furthermore, high positive interruption and weak belief in delay of gratification was significantly related to greater problem-solving pondering (see Figure 2c). Thus, Hypotheses 4 and 5 were supported, indicating three significant moderator effects on problem-solving pondering.

### Moderators: Hard work and self- reliance on detachment

As displayed in Tables S5 and S6, the interaction effect between positive attitudes towards work interruption as well as towards hard work (*ß* = 0. 11, *p*<0. 05) was significant for detachment, as was the interaction between positive interruption and self-reliance (*ß* = 0. 14, *p*<0. 01). The moderator effects are illustrated in Figure 2d, which shows that participants with low belief in hard work were less likely to detach themselves from work issues under the condition of high positive interruption, compared to those with strong belief in the importance of hard work. Further, employees with low belief in self-reliance (see Figure 2e) reported less detachment from work issues under high positive interruption compared to those with strong belief in self-reliance. Thus, Hypothesis6 is supported, indicating two significant moderator effects on detachment.

## Discussion

This study examined the influence of work beliefs and attitudes towards interruptions at work on psychological recovery post work. The results showed that problem-solving pondering was positively associated with work beliefs in morality/ethic, leisure, and the beliefs in centrality of work, and waste of time was negatively associated with problem-solving pondering. The results of the present research supported the Hypothesis 1.

### Linking work interruptions to problem-solving pondering: The role of wasted time and leisure

Hypotheses 2 and 3 concerning the mediating role of work beliefs in the relationship between positive/negative work interruption and problem-solving pondering were partially supported. First, the belief in the importance of efficient use of time was found to partially mediate the relationship between work interruptions and problem-solving pondering. Specifically, the ‘wasted time’ belief partially mediated the relationship between positive/negative appraisal of work interruption and problem-solving pondering, thus supporting Hypothesis 2. Strongly held beliefs in the importance of efficient use of time were associated with less frequent problem-solving pondering, better organization of time and higher work-engagement. This effect appears to be meaningful when a work interruption is perceived positively. Furthermore, it adds to the individual's ability to “switch-off” post work by becoming mentally detached after work tasks are completed on time. This is also consistent with previous findings in suggesting a beneficial impact on wellbeing by detaching from work during non-work time whilst remaining highly engaged at work [52], [42].

Interestingly, the current study supports the mediation effect of leisure beliefs in the relationship between positive appraisal of work interruption and problem-solving pondering. The mediating effect of leisure beliefs between positive interruption and problem-solving pondering is partial, suggesting the possibility of other work beliefs mediating the effect of positive appraisal of work interruption on problem-solving pondering independently of the belief in leisure.

The partial mediations found indicate that strongly held beliefs in the importance of leisure may trigger problem-solving pondering, whereas strongly held belief in efficient use of time may decrease problem-solving pondering. Nonetheless, the indirect mediated effects ranging from 0. 02 to 0. 03may be considered small. Research has regarded small effect sizes as important to the extent that the effect holds under different manipulations [53]. If this is so, then the effect is important not only because of the relationship between negative (0. 16) or positive (0.19) interruption and problem-solving pondering, but also because positive/negative work interruption may have a prolonged effect.

The fourth hypothesis, concerning the moderating role of work beliefs in the relationship between work interruptions and problem-solving pondering, was supported with reduced leisure-oriented attitudes as a potentially harmful moderator as it was associated with an increase in problem-solving pondering as well as negative evaluations of work interruptions. While weak belief in the centrality of work reduces problem-solving pondering under high negative interruption, strong belief in the centrality of work increases problem-solving pondering and heightens the appraisal of work interruptions as negative events.

The findings of the present research also support Hypothesis 5. Weak belief in delay of gratification increases problem-solving pondering under high appraisal of work interruptions as positive events. Hypothesis 6, concerning the moderating effect of beliefs in hard work and self-reliance on detachment from work, was also supported. Altogether, our findings suggest that individuals who value hard work per se and are self-reliant when completing work tasks are more likely to detach from work issues during off-job hours. Moreover, previous studies have found that detachment from work facilitates recovery including positive wellbeing, and prevents off-work psycho-physiological activation such fatigue, sleep problems, and need for recovery [35], [12],[11],[42].

The present research extends previous work on problem-solving pondering. Previous research argued that “creative” rumination is a functional cognitive process [27]. The present research found that positive attitude towards leisure was positively associated with increased problem-solving pondering, while the negative belief in wasted time was negatively associated with such rumination. Interruptions are a common workplace phenomenon and it was shown that employees who often fail to complete their work tasks are more likely to ruminate about work issues outside of work, leading to detrimental effects on recovery.

The current study highlights the importance of a number of work beliefs including beliefs about leisure and wasted time in problem-solving pondering. Specifically, it addresses two critical concerns: 1) the direct relationship between work interruptions and problem-solving pondering without consideration of the indirect influence of work beliefs. This implies that work interruptions contribute to ruminative thinking about problems at work; 2) and that work interruptions have an indirect effect through work belief in response to the appraisal of work interruptions as positive events. From this viewpoint, problem-solving pondering as a form of rumination is considered both an adaptive and a maladaptive cognitive process. As an adaptive cognitive process, employees tend to anticipate and solve problems during their leisure time when they have strong anti-leisure beliefs. They also perceive work interruptions as a positive challenge in relation to work goal achievement.

### Practical implications

The moderating role of the belief in centrality of work in the appraisal of work interruptions as negative events and problem-solving pondering has a number of implications. It is possible that centrality of work has different operationalisations that affect the moderating result. While some researchers conceptualise meaningful work as work related to task variety, feedback and autonomy [54], others define it as work that is intrinsically highly purposeful with job satisfaction independent of extrinsic rewards [55]. Future research may focus on defining the conceptualization of centrality of work and the construction of measurement scales for this work belief.

Another finding of our study is that detachment from work issues is more likely to occur when employees endorse strong beliefs in self-reliance and hard work combined with a positive appraisal of work interruptions. Generally, work interruptions are unavoidable and detachment during post-work time is essential.

Organization policies might initiate intervention programmes for employees whose work environment consists of constant interruptions: this could take the form of (1), time-management training, which would assist employees in completing work tasks; (2), assertiveness training, which would help employees to deal with work interruptions effectively, thereby they become more engaged in their work and to gain greater work satisfaction; and (3) detachment from work issues during off-job time (breaks, employee unavailability during off-job time).

The present study refers to a sample of business sector employees, therefore we cannot generalize the findings to employees of differing occupations, such as health care: Due to the nature of the job, employees working in health care for example, would have to cope with different types of work interruptions compared to the business sector, and such interruptions will probably be more urgent, more serious, and occur more frequently.

The use of the interruption scale highlights the importance of cognitive appraisals of the consequences of interruptions at work. As this is a new measure for assessing work interruptions, additional research is needed to test its validity. Further studies with different samples are necessary to replicate the current findings and to examine whether the interruption questionnaire items are invariant across occupations (managers, supervisors). Some researchers argued that “best practice” would occur if a measure were administered to an additional sample to assess the stability of the scale across time [57]. For this purpose, the test-retest reliability of the measure needs to be examined. While we have argued that our findings support the hypothesized relationships of work interruptions with work rumination through correlation and regression analysis conducted in the study, further demonstration of these relationships is important to support the validity of the new measure.

The size of the sample in the current study *(N = 310)* ensures that there is sufficient variance in responses, reducing any idiosyncratic concerns, and the sample of white collar employees of different occupational groups can be considered as a strength of the study. Given the partial mediation effect of beliefs in leisure, it would be crucial to consider how to prevent the negative effects of beliefs in leisure on problem-solving pondering. It may be argued that employees should make constructive use of leisure with pre-scheduled activities. A cognitive shift from the unproductive to constructive use of leisure could enhance positive emotions and promote better recovery. Arrangement of post-work leisure activities could include pursuing hobbies and learning new things, engagement in physical activities that reduce fatigue [56] or weekend socializing with others who share similar interests [29]. Social contacts during the weekend reinforce disengagement from work and promote wellbeing [29].

Although the current study does not derive causal conclusions, its findings suggest that negative attitude to wasted time is crucial for reducing problem-solving pondering, whereas positive belief in leisure reinforces problem-solving pondering. Evidence suggests that work detachment during leisure time is critical to regulate positive mood and to raise interest in work engagement [42]. However, there is a need for more longitudinal studies that shed light on the causal chain between work beliefs, work interruptions, problem-solving pondering and work detachment. While the balance between work and non-work life can replenish resources from work during non-work time, it is not always easy to attain. The use of emails and mobile devices may make it necessary to consider certain time slots of availability and norms of unavailability in order to help employees to recover from work interruptions [18]. For instance, daily time-interruption slots could be implemented to accommodate employees' complaints and needs.

## Conclusion

By studying work beliefs, our study has contributed to a better understanding of mechanisms fostering occupational recovery post work. The understanding of such relationships is critical to allow employees to ‘flow’ when dealing with work interruptions. The findings make important contributions. Firstly, they provide evidence that attitudes towards work interruptions are related to aspects of work rumination. And secondly, they expand our understanding of how employees detach from work. In sum, work beliefs are important determinants of a balanced work-leisure lifestyle that facilitates enhanced post work recovery and task performance.

## Supporting Information

Table S1 **Bivariate Pearson's r correlation coefficients between all study variables in the measures.**
(RAR)

Table S2 **Significant regression analyses for direct and indirect effects of positive and negative interruption on problem-solving pondering via anti-leisure and wasted time beliefs.**
(RAR)

Table S3 **Results of regression analysis of negative interruption and work beliefs on three types of work rumination.**
(RAR)

Table S4 **Results of regression analysis of negative interruption and work beliefs on three types of work rumination.**
(RAR)

Table S5 **Results of regression analysis of positive interruption and work beliefs on three types of work rumination.**
(RAR)

Table S6 **Results of regression analysis of positive interruption and work beliefs on three types of work rumination.**
(RAR)
